# Angioedema Shortly After Adding Empagliflozin to Losartan

**DOI:** 10.7759/cureus.78033

**Published:** 2025-01-26

**Authors:** Philip Duodu, Mina Aiad, Meriam Deeb, Mikaeel Jan, Dima Khalil, Melissa Wilson

**Affiliations:** 1 Hematology and Medical Oncology, St. Luke's University Health Network, Bethlehem, USA

**Keywords:** angioedema, angiotensin ii receptor blockers (arbs), empagliflozin, facial angioedema, hypersensitivity reactions, hypersensitivity vasculitis, losartan, losartan-induced angioedema, sglt, sglt-2 inhibitor

## Abstract

Angioedema is the localized swelling of the skin and mucosa caused by inflammatory processes that increase vascular permeability. Release and accumulation of the inflammatory mediators, bradykinin and histamine, result in fluid leakage from the plasma to the interstitium. Certain drugs, such as angiotensin-converting enzyme inhibitors (ACEi), promote bradykinin accumulation. In contrast, other allergens like insect bites, nuts, or non-steroidal anti-inflammatory drugs (NSAIDs) can cause histamine-mediated angioedema. While much less common than ACEi, angiotensin II receptor blockers (ARBs) are also associated with bradykinin-mediated angioedema. On the other hand, sodium-glucose cotransporter-2 (SGLT2) inhibitors, such as empagliflozin, are reported to cause rare hypersensitivity reactions present as angioedema, which are more known to be histamine-mediated.

Here, we present the case of a patient who developed angioedema three months after empagliflozin was added to her long-term losartan treatment, which she had been receiving for 39 months. To our knowledge, this is the first report of angioedema in the presence of SGLT2 and ARB inhibitors. This finding suggests a possible cumulative adverse effect of such medications and should be considered when prescribing both histamine and bradykinin effectors. Additionally, this case report serves as an example of the importance of the early diagnosis and treatment of angioedema.

## Introduction

Angioedema is a sudden, transient, and localized swelling of the dermis, subcutaneous tissue, mucosa, and submucosal tissues that can occur with or without urticaria. Airway obstruction can occur in severe cases. Hence, the cause of swelling must be identified quickly to reduce patient mortality. There are two main underlying pathophysiological mechanisms behind angioedema: histamine (or mast cell)-mediated and bradykinin-mediated. The latter, bradykinin-mediated angioedema, can be further classified into iatrogenic, acquired, and hereditary causes (e.g., C1 inhibitor deficiency). 

Bradykinin is an acute local inflammatory peptide that increases vascular permeability, vasodilation, bronchoconstriction, and pain. Drug-induced angioedema is caused primarily by angiotensin-converting enzyme inhibitors (ACEi). These drugs prevent the breakdown of bradykinin, leading to its accumulation and the exacerbation of its vasoactive effects. Around 25-39% of ACEi-induced angioedema cases present with life-threatening upper airway swelling; therefore, it is important to determine the patient's medication history and discontinue all ACEi [1]. Bradykinin-mediated angioedema does not respond well to epinephrine, antihistamines, or glucocorticoids [2]. 

In mast cell-mediated angioedema, mast cells release inflammatory mediators, including histamine, heparin, leukotriene C4, and prostaglandin D2, after being activated by foods, drugs such as antibiotics, non-steroidal anti-inflammatory drugs (NSAIDs), local anesthetics, stinging insects, or latex. These mediators cause the dilation of the venules in the dermis and increase permeability, resulting in edema [1]. Mast cell-mediated angioedema is associated with chronic urticaria. This type of angioedema can be treated with antihistamines and glucocorticoids.

## Case presentation

**Figure 1:**
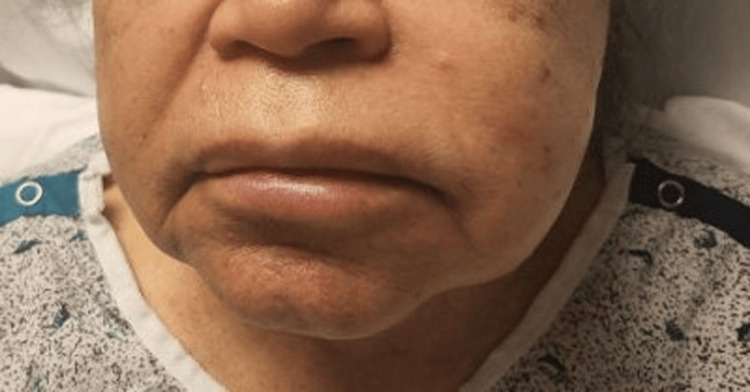
Angioedema of the left face.

An 82-year-old female patient with a history of type II diabetes mellitus and hypertension presented to the emergency department complaining of sudden-onset swelling of the left face and lips, with a tingling sensation (Figure 1). She could still swallow and protect her airway and denied shortness of breath or other symptoms. She had never experienced these symptoms before and denied any history of drug allergies or the use of new soaps, creams, cosmetics, or cleaning products. No hives were evident on the exam, and she denied pruritus. She was afebrile, with a heart rate range of 106-119 beats per minute, a systolic blood pressure ranging from 130s to 140s, and a respiratory rate of 18-22 breaths per minute. Her complete blood count, including white blood cell (WBC) differentials and the comprehensive metabolic profile, were all within normal limits.

**Figure 2:**
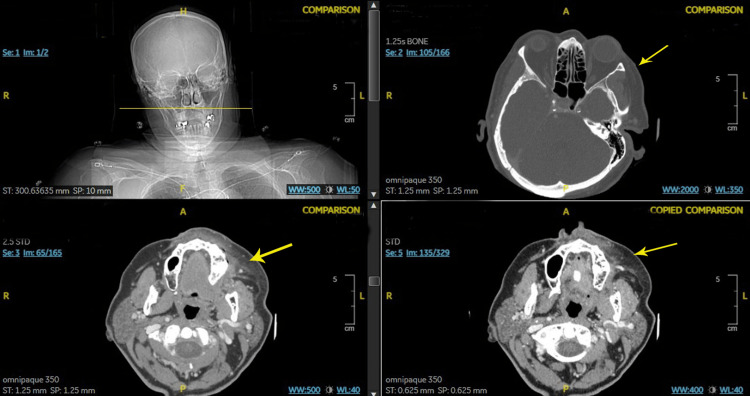
CT scan of the facial bones showing edema and stranding within the soft tissues and subcutaneous fat superficial to the left parasymphyseal mandible and maxilla (yellow arrows). CT: computed tomography

Given that angioedema was on top of her differentials, she received IV diphenhydramine 25 mg and IV methylprednisolone 125 mg. While the infection workup was in process, she was also given one dose of IV vancomycin, but later on, the two sets of blood cultures returned negative. The swelling rapidly improved within the next two hours. A computed tomography (CT) scan of the facial bone showed mild edema within the soft tissues and subcutaneous fat superficial to the left parasymphyseal mandible and maxilla, suggesting cellulitis (Figure 2). The medication review confirmed that she had been taking losartan 50 mg for 39 months and empagliflozin 25 mg for three months prior to symptoms but did not take any ACEi. The patient was diagnosed with drug-induced angioedema, and her angiotensin II receptor blocker (ARB) and sodium-glucose cotransporter-2 (SGLT2) inhibitors were both discontinued. She was discharged to home with oral prednisone and diphenhydramine. 

## Discussion

Angioedema is a serious drug-induced side effect that can result in airway obstruction owing to swelling of the dermis, subcutaneous tissue, and mucosal tissue. While ACEi are the most common cause of drug-induced angioedema, several other medications have been implicated in developing this reaction, including beta-lactam antibiotics and NSAIDs [1]. The underlying pathophysiology of angioedema is either due to a build-up of the peptide bradykinin, resulting in increased vascular permeability and vasodilation, or due to the release of the inflammatory mediator histamine from mast cells, resulting in vasodilation and increased permeability. It is essential to determine this difference in pathophysiology since bradykinin-mediated angioedema does not resolve following corticosteroid or antihistamine treatment, whereas histamine-mediated angioedema does. The etiology of bradykinin-mediated angioedema can be further classified into hereditary (C1 esterase inhibitor deficiency), acquired (lymphoproliferative and autoimmune disorders), and medication-induced (e.g., ACEi increases bradykinin levels) [3]. However, ACEi-induced angioedema is not entirely understood. Its management includes discontinuing ACEi and monitoring the airway. Glucocorticoids induce ACE expression and are thus expected to increase bradykinin metabolism, which may theoretically alleviate bradykinin-induced angioedema [4]. In acute attacks of hereditary and acquired bradykinin-induced angioedema, C1 inhibitor concentrate, kallikrein inhibitor ecallantide, bradykinin B2 antagonist icatibant, and fresh frozen plasma are used [2].

The case described here is a unique presentation of angioedema following the use of an ARB and SGLT2 inhibitor. In previous literature, ARBs have been implicated in bradykinin-mediated angioedema; however, the underlying mechanism is poorly understood. ARBs may result in the upregulation of angiotensin II and contribute to the development of angioedema [4]. In contrast, SGLT2 inhibitors cause rare histamine-mediated hypersensitivity reactions, including pruritus and angioedema [5]. 

Currently, it is not well understood whether bradykinin and histamine effector drugs can have cumulative effects when prescribed together. This case serves as a possible example of this effect since the patient had been taking an ARB for over three years and developed angioedema about three months after the addition of the SGLT2 inhibitor. Therefore, the patient's case may serve as a reason to be cautious before prescribing multiple medications associated with angioedema. It is unclear whether the angioedema in this case was due to the new drug, SGLT2 inhibitor, added to the patient's regimen or whether the addition resulted in a cumulative effect leading to angioedema. 

In addition, this case highlights the importance of the early diagnosis of angioedema. This physiological phenomenon's sudden and rapid progression can result in severe swelling of head and neck tissues, causing asphyxiation. The patient was managed with supportive measures, airway monitoring, and discontinuation of both ARB and SGLT2 inhibitors. Glucocorticoids and antihistamines were administered to cover the possible histamine-mediated swelling components. The effectiveness of treatment in reducing her swelling showcases the cruciality of early intervention. This report warrants caution in using medications with similar adverse events through separate mechanisms and the importance of close monitoring and recognizing possible adverse effects. 

## Conclusions

Angioedema can be a serious complication of certain medications. While ACEi, e.g., lisinopril, are the common inciting agents, other medications also are reported to cause angioedema, including ARBs, including losartan, as well as SGLT2 inhibitors, such as empagliflozin. The pathophysiology of the angioedema due to losartan is thought to be bradykinin-mediated. At the same time, empagliflozin causes histaminergic angioedema. Whether the use of two different medications associated with angioedema increases the risk of its occurrence or not requires more investigations and studies. The patient we are presenting developed angioedema three months after empagliflozin was added to her long-term losartan.

Increasing medical awareness of the medications that can cause angioedema, even those with less common incidence than ACEi and NSAIDs, is important for sooner diagnosis and quick management. Similarly, patients must be educated about the medications they use and all possible side effects, especially serious ones like angioedema. Discontinuing the offending agent is the first key step in drug-induced angioedema. Administering steroids and antihistaminic medications once the diagnosis is suspected is important for the quick resolution of the edema and decreases the risk of intubation. 

## References

[1] Bernstein JA, Moellman J (2012). Emerging concepts in the diagnosis and treatment of patients with undifferentiated angioedema. Int J Emerg Med.

[2] Ishigami K, Averill SL, Pollard JH, McDonald JM, Sato Y (2014). Radiologic manifestations of angioedema. Insights Imaging.

[3] Hébert J, Boursiquot JN, Chapdelaine H (2022). Bradykinin-induced angioedema in the emergency department. Int J Emerg Med.

[4] Brown T, Gonzalez J, Monteleone C (2017). Angiotensin-converting enzyme inhibitor-induced angioedema: a review of the literature. J Clin Hypertens (Greenwich).

[5] (2024). Jardiance. https://www.accessdata.fda.gov/drugsatfda_docs/label/2022/204629s033lbl.pdf.

